# Broth *versus* Surface-Grown Cells: Differential Regulation of RsmY/Z Small RNAs in *Pseudomonas aeruginosa* by the Gac/HptB System

**DOI:** 10.3389/fmicb.2016.02168

**Published:** 2017-01-10

**Authors:** Fabrice Jean-Pierre, Julien Tremblay, Eric Déziel

**Affiliations:** ^1^Institut National de la Recherche Scientifique, Institut Armand-Frappier, LavalQC, Canada; ^2^National Research Council Canada, MontréalQC, Canada

**Keywords:** *swarming*, broth-surface, small RNAs, surface motility, genetic regulation, histidine phosphotransfer protein, HptB

## Abstract

Two-component systems are capable of profoundly affecting genetic regulation in bacteria by detecting environmental stimuli, allowing them to quickly adapt. In *Pseudomonas aeruginosa*, the small RNAs (sRNAs) RsmY and RsmZ are under the control of the GacS/A system. They have been described as ones of the major key players in the control of planktonic and surface-associated behaviors. Genetic regulation by these sRNAs is achieved by the titration of the negative post-transcriptional regulator RsmA which affects the expression of over 500 genes. There is increasing evidence pinpointing the importance of RsmY and RsmZ in the planktonic-sessile *P. aeruginosa* lifestyles switch control. Using swarming motility as a model, we show here that these sRNA are differentially regulated depending on the selected growth conditions (i.e., planktonic *versus* surface grown-cells). Also, we report that opposite to planktonically grown cells, *rsmZ* regulation does not implicate the response regulator GacA in swarming cells. Furthermore, we present data indicating that RsmY/Z expression influence swarming motility *via* the protein HptB which acts as a negative regulator of these sRNAs and that they do not strictly converge to RsmA as previously reported.

## Introduction

Bacterial survival in the environment relies on their capacity to quickly adapt to changing conditions by either inducing or repressing specific sets of genes (Boor, 2006). Some bacteria have the ability to colonize a broad range of hosts using virulence functions (Furukawa et al., 2006). One adaptation mechanism used by bacteria consist of two-component systems (TCSs) (Hoch and Varughese, 2001), membrane-bound sensors coupled to cytoplasmic response regulators that permit the integration of external stimuli and induce global gene expression shifts (Beier and Gross, 2006). The heterotrophic opportunistic pathogen *Pseudomonas aeruginosa* exemplifies such a remarkable capacity to adapt to changing environments by encoding more than 60 TCS on its genome (Rodrigue et al., 2000). The GacS/GacA TCS has been extensively described over the years and is central in regulating the expression of the two small RNAs (sRNAs) RsmY and RsmZ. Mainly controlled by the response regulator GacA, these sRNAs titrate the availability of RsmA, a post-transcriptional regulator modulating the expression of functions implicated in the transition between the surface-associated and the planktonic *P. aeruginosa* lifestyles (Hoch and Varughese, 2001; Brencic and Lory, 2009; Brencic et al., 2009).

Swarming motility is a surface-associated type of bacterial motility characterized by a rapid and coordinated movement of a bacterial population on a viscous surface (Kearns, 2010). In *P. aeruginosa*, this type of bacterial movement necessitates the presence of functional flagella and the production of rhamnolipids (and other RhlABC products) responsible for both modulation of the surface behavior and lowering surface tension (Déziel et al., 2003; Tremblay et al., 2007). Over the years, swarming motility has been linked to many phenotypes, such as increased antibiotic resistance, and to be inversely regulated to another surface bacterial behavior, biofilm formation (Caiazza et al., 2007; Lai et al., 2009). Given that swarming motility represents a distinct bacterial lifestyle from planktonic and sessile cells, many studies have aimed at understanding how this surface behavior is regulated. Over the years, the secondary messenger molecule, c-di-GMP has been shown to have a profound impact over the motile-sessile behavior in the bacterial kingdom, but in *P. aeruginosa* its implication in swarming motility is still not completely clear yet and is a matter of active research as some contradictions have been reported (Caiazza et al., 2007; Kuchma et al., 2007; Merritt et al., 2007; Baker et al., 2016). Excluding the impact of secondary messengers, this surface behavior is still poorly understood at the expression level. In an attempt to better characterize swarming motility, Overhage et al. (2008), defined how this surface behavior was affected and observed that many virulence genes were upregulated in swarming cells compared to their planktonic (free-swimming) counterpart. To further dissect and understand the complexity of swarming motility, we have previously (Tremblay and Déziel, 2010) conducted a transcriptomic analysis of swarming cells compared to bacteria that were grown on the same agar-solidifed medium just dried longer to prevent swarming motility (surface *versus* surface). In contrast with Overhage et al. (2008), we found that cells at the migrating tip of a swarming colony downregulated virulence factor expression but upregulated genes associated with energy metabolism. The apparent discrepancies between the two studies were explained by differences in experimental design. Indeed, Overhage et al. (2008), compared cells at the swarm tip against planktonic cells (surface *versus* broth) whereas Tremblay and Déziel (2010) compared surface *versus* surface. The observation of such opposite results between these two studies also raised the possibility of the existence of unknown regulation pathways specific to surface-grown cells, here in the context of swarming motility.

The study of swarming motility at the regulatory level is complex given that two critical factors, namely flagellar function and biosurfactants production, are imperative for that surface behavior. Thus, to better understand how swarming motility is regulated, it is helpful to identify mutants incapable of such a type of social movement while still possessing a functional flagellum and with no defect in production of rhamnolipids. Interestingly, a mutation in the *hptB* gene, encoding for one of the three histidine phosphotransfer proteins implicated in signal transduction in *P. aeruginosa*, was reported to negatively affect swarming motility (Hsu et al., 2008). A microarray analysis of a Δ*hptB* mutant has shown that many flagellar-related genes are affected but surprisingly, no defect in swimming motility was observed (Bhuwan et al., 2012). Also, the production of rhamnolipids was never addressed in these studies, thus not covering all the possibilities as to why such a mutant is incapable of swarming motility. Furthermore, HptB was reported to interact with hybrid sensor kinases, one of which is RetS implicated in the control of the GacS/GacA system to affect global gene expression (Goodman et al., 2004; Lin et al., 2006; Brencic et al., 2009; Bordi et al., 2010). Interestingly, Bordi et al. (2010) determined that HptB mediates its effect through the modulation of the sRNA RsmY exclusively.

In the present study, we demonstrate how HptB affects swarming motility *via* modulation of the expression of both RsmY and RsmZ and that the swarming default of an Δ*hptB* mutant is not associated with flagellar malfunction nor insufficient biosurfactant production. Comparing Δ*hptB* mutant cells cultivated in planktonic *versus* surface conditions, we show that both *rsmY* and *rsmZ* are negatively regulated by HptB. Furthermore, HptB-mediated *rsmZ* expression control is stronger in swarming cells and, unexpectedly, not under the absolute control of the GacA response regulator.

## Materials and Methods

### Strains, Plasmids, and Growth Conditions

Bacteria used in this study are all derived from the parental strain PA14 (Rahme et al., 1995; Lee et al., 2006) and are listed in **Table 1**. Bacteria were cultivated at 37°C in Tryptic Soy Broth (TSB) (Difco) with shaking (240 rpm) in a TC-7 roller drum (New Brunswick) or on TSB plates solidified with 1.5% agar. For swarming motility assays, overnight cultures were washed in phosphate-buffered saline (PBS) and diluted to the desired OD_600_. For transcriptomic analyses, the bacteria were cultivated in M9 minimal medium supplemented with 11 mM dextrose and 0.5% casamino acids (Difco) (M9DCAA) broth at 34°C (Tremblay and Déziel, 2008). OD_600_ was measured with a Nanodrop ND-1000 (Thermo Fisher Scientific) and pathlength correction was applied by multiplying the given value by a factor of 10.

**Table 1:** Strains/plasmids used in this study.

Strains/plasmids	ED #	Phenotype/genotype	Reference
***E. coli***			
DH5α	78	*fhuA2 Δ(argF-lacZ)U169 phoA glnV44 Φ80 Δ(lacZ)M15 gyrA96 recA1 relA1 endA1 thi-1 hsdR17*	Woodcock et al., 1989
SM10 (λpir)	222	*thi thr leu tonA lacY supE recA::RP4-2-Tc::Mu Km^R^ λpir*	Simon et al., 1983
***P. aeruginosa***			
PA14	14	UCBPP-PA14 wild-type strain	Rahme et al., 1995
Δ*hptB*	1214	Markerless deletion of *hptB* in PA14	This study
Δ*rsmY*	1971	Markerless deletion of *rsmY* in PA14	Laboratory collection
Δ*rsmZ*	1976	Markerless deletion of *rsmZ* in PA14	Laboratory collection
Δ*rsmYZ*	1998	Markerless deletion of *rsmY* and *rsmZ* in PA14	Laboratory collection
*gacA^-^*	1800	*MrT7* transposon mutant ID 34781 Gm^R^	Liberati et al., 2006
Δ*hptBgacA*	2654	Markerless *hptB* deletion in PA14, *gacA::MrT7* transposon mutant ID 34781 Gm^R^	This study
Δ*hptBrsmY*	2831	Markerless deletion of *hptB* and *rsmY* in PA14	This study
Δ*hptBrsmZ*	2832	Markerless deletion of *hptB* and *rsmZ* in PA14	This study
Δ*hptBrsmYZ*	2833	Markerless deletion of *hptB, rsmY* and *rsmZ* in PA14	This study
**Plasmids**			
pEX18Tc		Suicide vector in *P. aeruginosa SacB*, Tc^R^	Hoang et al., 1998
pJT39	1306	Suicide vector containing the *hptB* gene with an absent histidine phosphotransfer domain	This study
pEXG2-Δ*rsmY*		Suicide vector for Δ*rsmY* mutation construction	Brencic et al., 2009
pEXG2-Δ*rsmZ*		Suicide vector for Δ*rsmZ* mutation construction	Brencic et al., 2009

### Construction of the Δ*hptB* Knock-Out Mutant

Deletion of the Hpt domain of the *hptB* gene (PA3345 in PAO1 and PA14_20800 in PA14) was performed as follows. Two fragments of DNA were amplified from PA14 genomic DNA using pairs of primers PA3345_Left_FWD and PA3345_Left_REV for the left fragment and PA3345_Right_FWD and PA3345_Right_REV for the right fragment. These fragments were ligated together using a complementary engineered overhang sequences of 15 nucleotides common to PA3345_Left_REV and PA3345_Right_FWD. The DNA fragment was then cloned in the MCS of pEX18Tc using restriction enzymes HindIII and SmaI. The resulting plasmid (pEX18Tc_Δ*hptB*) was transformed into *Escherichia coli* SM10 on TSB plates containing 15 μg ml^-1^ of tetracycline (Tc). A two partner conjugation between PA14 and SM10-pEX18Tc_Δ*hptB* was then performed. Tetracycline resistant simple recombinants were selected on tetracycline 125 μg ml^-1^ TSB plates and streaked onto TSB without antibiotic overnight. Double recombinants were selected by plating these overnight grown cells on LB (without NaCl) supplemented with 8% sucrose. Positive clones were confirmed by polymerase chain reaction (PCR) and sequencing.

### Motility Assays

Swarming motility assays were performed using the same medium as for swimming motility tests but with 0.5% agar. Once autoclaved, the semi-solid agar was dried in a laminar biological safety cabinet for 75 min. Swarming plates were inoculated with 5 μl of a bacterial subculture grown in TSB to early stationary phase and adjusted to an OD_600_ of 3.0 in sterile 1X PBS. The plate were then incubated overnight at 34°C (Tremblay and Déziel, 2008). Time-lapse image analysis was done using Photoshop CS3 Extended (Adobe).

Swimming motility plates were done by inoculating 3 μl of bacterial suspension at an OD_600_ of 3.0 directly inside LB or M9DCAA plates solidified with 0.25% agar. The swimming diameter was measured after overnight incubation at 34°C. Experiments for testing swimming and swarming motility were performed with a minimum of three to five technical replicates on two separate days. Statistical analysis was done using Prism version 6.0 (GraphPad).

### RNA Preparation

Total RNA was extracted from liquid bacterial cultures grown in triplicate in M9DCAA cultivated to late exponential phase (OD_600_ = 1.3) at 34°C. The cells were then centrifuged for 5 min at 12,000 × *g* and the supernatant was discarded. Cells were resuspended in PureZOL (BioRad) and RNA extraction was performed following the manufacturer’s recommendations. For surface-grown bacteria, RNA was collected from the cells located at the migrating tip of a swarming colony grown for 12 h at 34°C using 8 μl of RNAlater (Qiagen) that was put directly on each tendril tip, as previously described (Tremblay and Déziel, 2010). Cells were resuspended by pipetting and transferred to a 1.5 ml microcentrifuge tube kept on ice. An average of 8 migrating tendril tips were harvested per plate with three plates for each biological replicate for a total of nine plates. RNA extraction was performed using PureZOL.

### Quantitative Real-Time PCR (qRT-PCR)

Quantitative real-time PCR (qRT-PCR) was done with the *qScript*^TM^ *One-Step SYBR Green* kit and a RotorGene 6000 (Corbett) thermocycler. Primers were designed in order to obtain amplicons of 80–150 bp^1^. The *nadB* gene was used as control. Each cycle of qRT-PCR was done in triplicate. The threshold cycle (*C*t) was normalized to *nadB C*t amplified in each corresponding samples. Variation in expression was calculated using the -2^ΔΔCt^ method (Livak and Schmittgen, 2001). Assessment of variation in expression was performed using biological triplicates on two different days. Statistical analysis was done using Prism version 6.0 (GraphPad). Gene expression variation is shown as Relative expression variation (log_2_) to the wild-type PA14 strain.

### LC/MS Rhamnolipids Quantification

Concentrations of rhamnolipids were determined by liquid chromatography(LC)/mass spectrometry (MS) (Abdel-Mawgoud et al., 2014). A 400 μl sample of a liquid overnight culture was collected and the OD_600_ was measured. Then the samples were centrifuged at 16,000 × *g* for 10 min. to remove bacterial cells. To 300 μl of supernatant, 300 μl acetonitrile containing 20 mg/L 5,6,7,8-tetradeutero-4-hydroxy-2-heptylquinoline (HHQ-d4) as the internal standard were added. Samples were analyzed by high-performance liquid chromatography (HPLC; Waters 2795, Mississauga, ON, Canada) equipped with a C8 reverse-phase column (Kinetex, Phenomenex) using a water/acetonitrile gradient with a constant 2 mmol l^-1^ concentration of ammonium acetate. The detector was a mass spectrometer (Quattro Premier XE, Waters). Analyses were carried out in the negative electrospray ionization (ESI-) mode.

Surface biosurfactant production was measured by inoculating the Δ*hptB* mutant and the wild-type strain on swarm plates. After overnight incubation at 34°C, all the agar was recovered from the Petri dishes and transferred to an Erlenmeyer flask containing 1:1 (v/v) KHCO_3_ (pH 9.0) (i.e., for five 20 ml swarm plates, 100 ml of KHCO_3_ was added). The collected agar was vigorously mixed for 1 h at room temperature then filtered through Whatman no. 1 covered with silica sand (Fisher). The filtrate was acidified at pH 4.0 with concentrated HCl then extracted three times with 50 ml ethyl acetate. The organic phases were pooled, evaporated and the residue analyzed as described above. Total biomass was quantified by resuspending bacteria on the swarm plates twice with 1 ml PBS then transferred to pre-weighed aluminum boats, dried at 65°C for 2 h and then weighed again. Biomass was measured by preparing a parallel swarm set in identical conditions for rhamnolipids quantification. Rhamnolipids quantification was done in technical triplicate on two different days. Statistical analysis was done using Prism version 6.0 (GraphPad).

## Results

### HptB-Mediated Swarming Regulation Does Not Implicate Flagellar Malfunction nor a Default in Rhamnolipids Production

**FIGURE 1:**
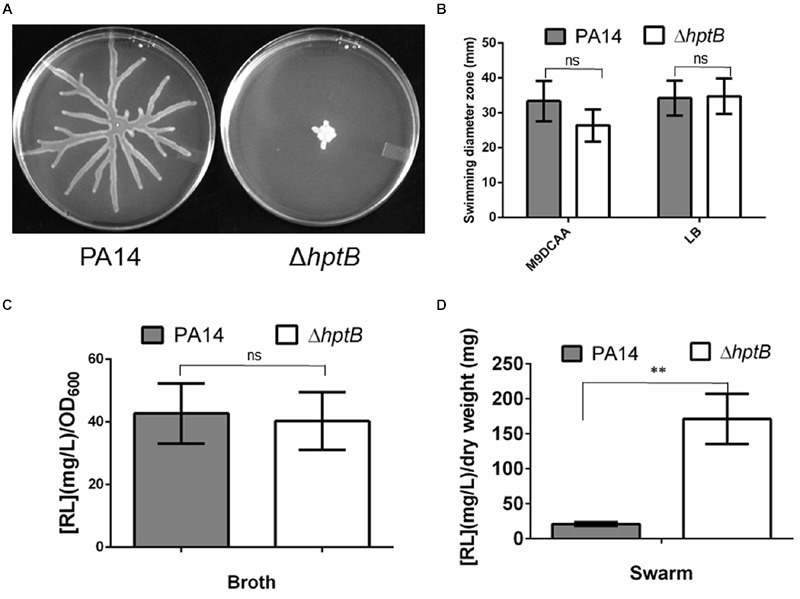
**A mutation in the *hptB* gene affects swarming motility. (A)** Swarming motility phenotypes of the wild-type PA14 strain and the Δ*hptB* mutant. **(B)** Swimming motility assay in various media containing 0.25% agar. **(C)** Rhamnolipids quantification in planktonic cultures. **(D)** Rhamnolipids quantification on swarming plates. Error bars represent the standard error of the mean for experiments carried out at least twice with a minimum of three replicates per experiment. Student’s *t-*test analysis was applied on two independent experiments with ^∗∗^*p* < 0.01; ns, not significant.

There is increasing evidence for the implication of the HptB phosphotransfer protein in the modulation of several *P. aeruginosa* behaviors (Hsu et al., 2008; Bordi et al., 2010; Kong et al., 2013). Recently, swarming motility has been identified as being under the control of the HptB regulon, which was determined to include flagella-related genes (Bhuwan et al., 2012); however, surprisingly, no effect was seen on flagellar functionality. To further characterize the role of HptB in the regulation of the swarming surface-associated behavior, we engineered a markerless Δ*hptB* mutant by deleting the entire histidine phosphotransfer domain (Hsu et al., 2008). As previously noted by Hsu et al. (2008), for strain PAO1, inactivation of the *hptB* gene in strain PA14 results in the loss of swarming motility (**Figure 1A**). A functional flagellum and the production of RhlABC products (mostly rhamnolipids) are elements required for swarming motility (Köhler et al., 2000; Déziel et al., 2003; Caiazza et al., 2005; Tremblay et al., 2007). Thus, we verified whether or not flagellar functionality is responsible for the Δ*hptB* mutant swarming phenotype by performing swimming motility assays. As shown in **Figure 1B**, the Δ*hptB* mutant still has a functional flagellum in both tested conditions. The production of rhamnolipids was also verified both in planktonic cultures and directly on swarm plates. The Δ*hptB* mutant produces an equivalent amount of biosurfactant compared to the wild-type strain when incubated in M9DCAA broth (**Figure 1C**). When looking at production of rhamnolipids by swarming cells directly from plates the Δ*hptB* mutant is not impaired and it actually displays increased biosurfactant production (**Figure 1D**). Absence of a growth defect by the Δ*hptB* mutant (Supplementary Figure S1) excludes known factors for the observed deficient swarming phenotype. We conclude that HptB positive control on swarming motility does not go through the known required factors.

### Swarming Motility is Linked to sRNAs Expression

**FIGURE 2:**
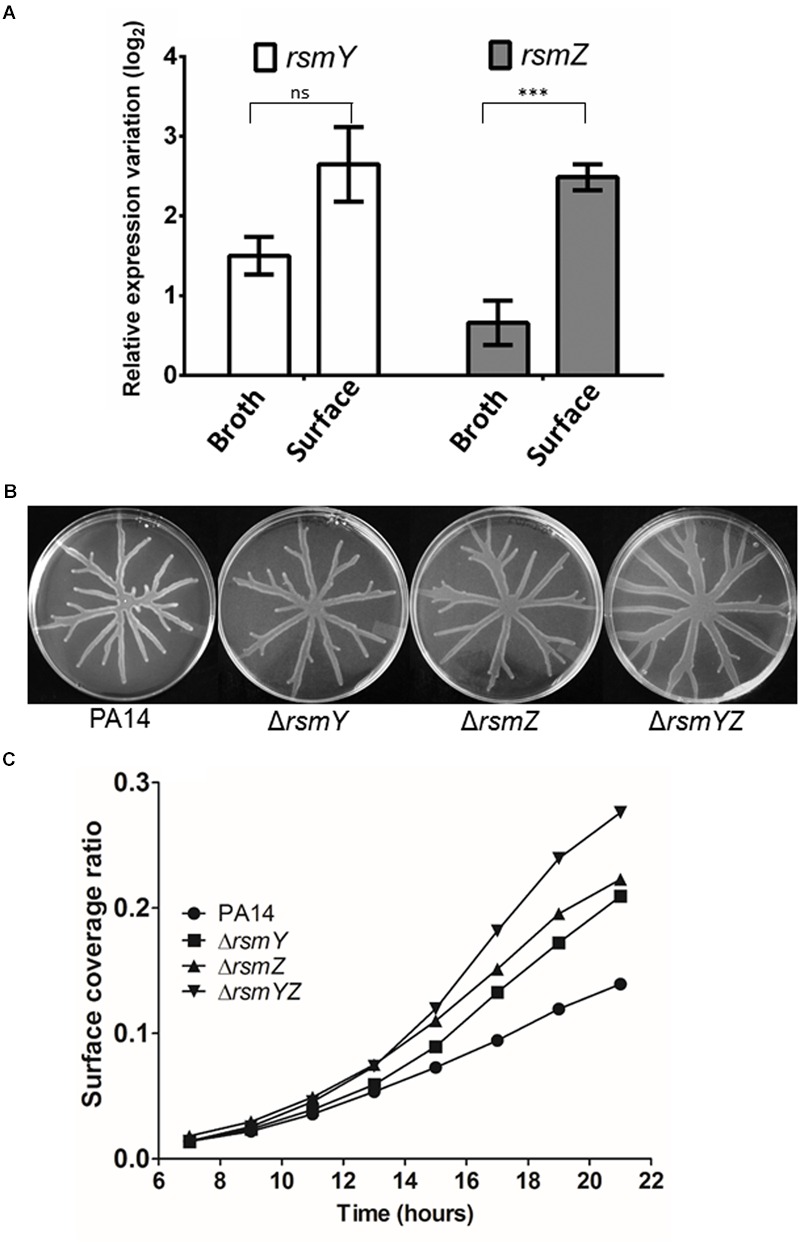
**Swarming motility is linked to sRNA expression. (A)** Expression of the sRNAs RsmY and RsmZ in the Δ*hptB* mutant strain grown in broth or on a surface (swarming condition) determined by qRT-PCR. **(B)** Swarming motility of various sRNA mutants. **(C)** Time-lapse analysis of the Δ*rsmY/Z* mutants. Error bars represent the standard error of the mean for experiments carried out at least twice with three biological replicates per experiment. Student’s *t-*test analysis was applied on two independent experiments with ^∗∗∗^*p* < 0.001; ns, not significant. For **(C)**, the data correspond to one single plate per strain. Gene expression variation is shown as relative expression variation (log_2_) to the wild-type PA14 strain.

There are indications that expression of the swarming phenotype is positively regulated by RsmA and antagonized by the small RNAs, RsmY and RsmZ (Heurlier et al., 2004; Kay et al., 2006). In cells cultivated as broth cultures, HptB negatively affects the expression of RsmY specifically, but with no effect on RsmZ (Bordi et al., 2010). Since bacterial growth mode (i.e., cells grown planktonically *versus* on a surface) can affect the output of genetic regulation (Overhage et al., 2008; Tremblay and Déziel, 2010), we monitored the expression of both *rsmY* and *rsmZ* in bacteria grown in both planktonic and swarming cultures. In agreement with what was seen in strain PAK (Bordi et al., 2010), the RsmY sRNA is also overexpressed in the PA14 Δ*hptB* mutant grown in broth cultures while a small (and not previously reported) increase in expression of RsmZ is also observed (**Figure 2A**). Surprisingly, when the same experiment is carried out on swarming cells sampled at the migrating tip of a swarming colony, a marked increase of 2 log_2_ in *rsmZ* expression is observed when compared to its planktonic counterpart (**Figure 2A**), suggesting specific upregulation by surface growth. We therefore looked at the swarming phenotypes of Δ*rsmY*, Δ*rsmZ*, and Δ*rsmYZ* mutants and observed that they all exhibit a hyperswarming phenotype (**Figure 2B**), when compared to the wild-type (**Figure 1A**). To precisely confirm this hyperswarmer behavior, we measured the area covered by the swarming colony using time-lapse image analysis: Δ*rsmY* and Δ*rsmZ* behave the same way while the increased swarming phenotype of the double mutant is even more pronounced than both simple mutants (**Figure 2C**; Supplementary Figure S2).

**FIGURE 3:**
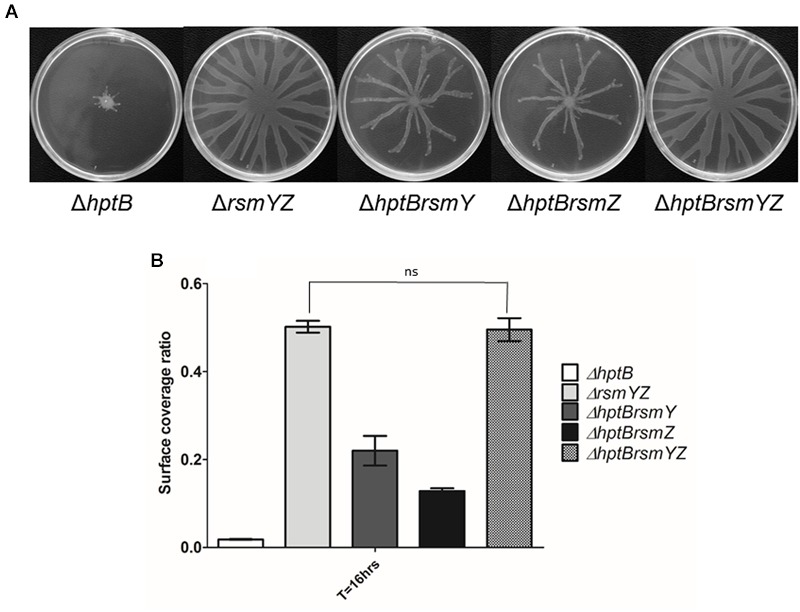
**Swarming deficiency of the Δ*hptB* mutant is due to sRNA overexpression. (A)** Swarming motility assessment of various double and triple mutants. Shown are the average representative swarming phenotype of various strains. **(B)** Swarming motility surface coverage of the simple Δ*hptB* mutant, the double Δ*hptBrsmY/Z* mutants, the triple Δ*hptBrsmYZ* mutant and Δ*rsmYZ* double mutant. Student’s *t-*test analysis was done on the Δ*rsmYZ* double mutant and the triple Δ*hptBrsmYZ* mutant based on two independent experiments (ns, not significant). Error bars represent the standard deviation of three technical replicates.

To verify the hypothesis that the swarming defect of the Δ*hptB* mutant is directly caused by the overexpression of *rsmY* and *rsmZ*, we constructed double Δ*hptBrsmY*, Δ*hptBrsmZ* and triple Δ*hptBrsmYZ* mutants. Significantly, the introduction of either a *rsmY* or a *rsmZ* mutation in a Δ*hptB* background resulted in rescue of the swarming phenotype, yet not identical as the pattern observed for the simple Δ*rsmY* and Δ*rsmZ* mutants (**Figure 3A**). Finally, swarming motility of the triple Δ*hptBrsmYZ* exhibited exactly the same phenotype as the double Δ*rsmYZ* (**Figure 3B**). Taken together, these results support a model where HptB promotes swarming motility through the RsmA pathway, likely by decreasing the expression of both inhibitory sRNAs RsmY and RsmZ.

### Regulation of *rsmZ* Differs in Surface-Grown Cells Compared to their Planktonic Counterpart

**FIGURE 4:**
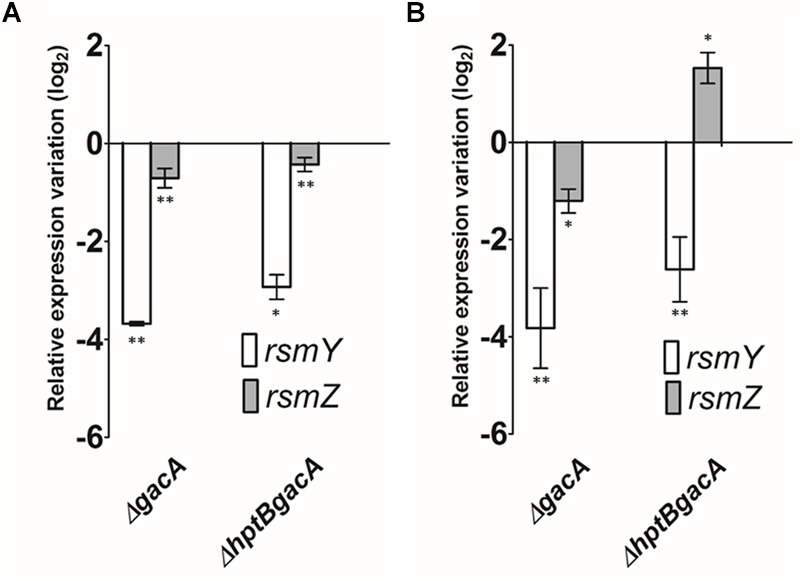
**The expression of sRNA is dependent of growth conditions. (A)** Expression of *rsmY* and *rsmZ* determined by qRT-PCR in various genetic backgrounds grown in broth. **(B)** Expression of *rsmY* and *rsmZ* of strains grown on a surface (swarming). Error bars represent the standard deviation of experiments carried out using three biological replicates. Student’s *t-*test analysis was based on two independent experiments (^∗^*p* < 0.05; ^∗∗^*p* < 0.01). Gene expression variation is shown as relative expression variation (log_2_) to the wild-type PA14 strain.

To understand if the regulation of *rsmY* and *rsmZ* by HptB is exerted through the GacS/GacA TCS, which has been characterized as being responsible for the transcription of these two sRNAs in liquid cultures (Brencic et al., 2009), we engineered a double Δ*hptBgacA* mutant (Supplementary Material). In that mutant background, we monitored the expression of *rsmY* and *rsmZ* in planktonic and surface-associated (swarming) cells. When the cells are cultivated in broth, the expression levels of both *rsmY* and *rsmZ* are indeed negatively affected in the double Δ*hptBgacA* in a comparable way to the simple Δ*gacA* mutant, supporting the hypothesis that the control of HptB over *rsmY* and *rsmZ* occurs downstream of GacA (**Figure 4A**). Similarly, when the same experiment was performed on swarmer cells (surface-growing bacteria), a similar negative effect of HptB on *rsmY* was confirmed to be mediated through GacA. On the other hand, unexpectedly, the overexpression of *rsmZ* that was seen in the Δ*hptB* mutant background (**Figure 2A**) is still observed when *gacA* is also abrogated (**Figure 4B**), indicating that HptB regulates *rsmZ* expression via an unidentified alternative regulatory pathway independent of GacA, specific to bacteria growing on a surface.

## Discussion

Swarming motility is a complex multicellular phenomenon that has been extensively investigated over the years. However, the underlying genetic regulatory pathways controlling that surface-associated type of motility still remain to be fully characterized in *P. aeruginosa*. In the present study, we have identified that the inactivation of the *hptB* gene renders *P. aeruginosa* incapable of such a type of motility even though this mutant still expresses the necessary propelling and wetting tools to exert a normal movement on a semi-solid (0.5% agar) medium.

The HptB protein has been well-described and novel pathways implicating this protein have been identified. Bhuwan et al. (2012), saw that transcription of many flagella-related genes was affected in a Δ*hptB* mutant grown as swarming cells and that these effects were mediated via a novel regulatory cascade implicating the *PA3346* and *PA3347* gene products. Surprisingly, they reported no differences in flagellar morphology and swimming motility of the Δ*hptB* mutant, indicating that the functionality of this propelling appendage was apparently not affected. Thus, a regulatory imbalance in that mutant was possibly responsible for such a phenotype. However, Lin et al. (2006), observed that the absence of HptB provoked an important decrease in swimming motility in the PAO1 strain. In contrast to these studies, we looked at flagellar functionality and did not observe a defect in swimming motility of the Δ*hptB* mutant that could explain the dramatic decrease in swarming motility. The discrepancies observed between these two studies and ours could be due to differences in experimental design and strains. Bhuwan et al. (2012), incubated their swarming plates at 30°C for 36 h before proceeding to their transcriptomic analyses. Furthermore, they looked at their swarming phenotypes by incubating their plates at 37°C for 36 h. Our experiments were all carried out using plates incubated at 34°C for 12–16 h, when the cells are still metabolically active (Tremblay and Déziel, 2010).

Also, previous studies looking at the implication of HptB in motility never addressed the question of RhlABC products (biosurfactants). To express the swarming phenotype, *P. aeruginosa* needs the production of the wetting agent rhamnolipids (Déziel et al., 2003; Caiazza et al., 2005). A Δ*rhlA* mutant is incapable of swarming motility thus looking at the production of the biosurfactants is imperative in studies investigating this type of surface-associated motility. Here, we looked at the production of rhamnolipids in the Δ*hptB* mutant and did not see differences in production compared to the wild-type strain when the cells were cultivated in liquid cultures. Interestingly the same mutant produced more rhamnolipids than wild-type PA14 under swarming conditions. We hypothesize that rhamnolipid production is upregulated to overcome the absence of swarming. Accordingly, a 1:1 co-culture of the Δ*hptB* and *rhlA^-^* mutants results in a rescue of swarming motility of a Δ*rhlA* mutant strain (data not shown) and therefore we have no reason to believe that overproduction of these wetting agents would prevent such a type of motility, quite the opposite. Thus, *hptB* was considered an interesting gene to investigate how swarming motility is regulated.

The membrane sensor RetS is capable of phosphorylating the HptB protein (Hsu et al., 2008). Transcriptomic analyses performed on planktonic bacteria have revealed that the HptB and RetS regulons are partially overlapping but consist of two separate signaling pathways that both converge to the GacS/GacA system through different mechanisms (Bordi et al., 2010). Interestingly, HptB was seen to have an effect on the regulation of the small RNA RsmY specifically and implicated an alternative pathway including the *PA3346* and *PA3347* gene products whereas no effect of the phosphotransfer protein was observed on RsmZ (Bordi et al., 2010). Thus, since HptB seems to control many phenotypes *via* RsmY regulation we investigated the effect of a Δ*hptB* mutation on small RNA regulation in both planktonic and swarming cells and confirmed the increased transcription of *rsmY* in both conditions at the same extent. However, in contrast with that study, we observe a moderate increase in *rsmZ* expression in a planktonic culture (**Figure 2A**). Since swarming is a surface-associated bacterial behavior, we also looked at the expression of both *rsmY* and *rsmZ* on cells that were collected at the tip of a migrating colony. Unexpectedly, we observed a 2 log_2_ increase in expression of *rsmZ* in swarming cells compared to their planktonic counterparts. This result indicates that *rsmZ* is differently regulated when cells are grown on a surface specifically. Such a different regulation on sRNAs is not unusual. For instance, Petrova and Sauer (2010) found that the inactivation of *bfiS*, implicated in biofilm formation, resulted in increased *rsmY* and *rsmZ* expression strictly in cells cultivated as biofilms but not in planktonic ones. Here, we found only *rsmZ* to be upregulated when comparing cells cultivated in broth *versus* a surface. This intriguing result guided us toward asking whether RsmZ could be the main mediator of swarming motility. Thus, we decided to look at the swarming phenotype of the simple and double Δ*rsmY/Z* mutants. We expected to see an effect on swarming only for the Δ*rsmZ* mutant. Contrary to what has been observed by other groups (Heurlier et al., 2004; Kay et al., 2006), we saw an increase in swarming motility of both single mutants. The inactivation of both genes resulted in an even better capacity to swarm (**Figures 2B,C**). These results indicate that both RsmY and RsmZ act as negative regulatory elements of swarming motility and that the observed surface motility defect of the Δ*hptB* mutant is explained by the overexpression of these two sRNA. As a matter of fact, we have also observed that the overexpression of either RsmY or RsmZ in the wild-type PA14 background results in a decrease in swarming motility (data not shown). Interestingly, other reporter phenotypes such as increased exopolysaccharide production and hyperbiofilm formation seen in the PAKΔ*hptB* (Bordi et al., 2010) was not detected in a PA14Δ*hptB* mutant (Supplementary Figures S3 and S4), indicating that these strains behave differently, as previously reported for swarming motility for instance (Tremblay and Déziel, 2008).

To associate without any further doubt the implication of HptB in the regulation of swarming motility *via* the downregulation of both *rsmY* and *rsmZ*, we created a triple Δ*hptBrsmYZ* mutant. Swarming motility assay of that mutant resulted in a complete rescue of the phenotype equivalent to that of the double Δ*rsmYZ* strain. Swarming assessment of both double Δ*hptBrsmY* and Δ*hptBrsmZ* mutants somehow resulted in an intermediate surface motility phenotype, further confirming that both sRNAs are important for this type of surface-associated movement and have a cumulative effect. Furthermore, Bordi et al. (2010) demonstrated a corresponding sRNA summative effect, as the abolishment of either *rsmY* or *rsmZ* in a PAKΔ*hptB* mutant background resulted in the production of intermediate biofilm phenotypes, whereas a triple Δ*hptBrsmYZ* mutant strain behaved exactly like a double Δ*rsmYZ* mutant. Together, these findings validate that the investigated phenotypes affected by the deletion of the *hptB* gene (in PA14 and PAK strains) are linked to *rsmY* and *rsmZ* overexpression.

Interestingly, an *rsmA^-^* mutant is not as defective in swarming motility as the Δ*hptB* mutant (Supplementary Figure S5). Also, the inactivation of *rsmA* leads to impairment of rhamnolipids synthesis thus explaining its incapacity to swarm properly (Heurlier et al., 2004) while it is not the case for the Δ*hptB* mutant. Recently, the novel post-transcriptional regulator, RsmN (an RsmA ortholog) has been described as a positive regulator of swarming motility (Morris et al., 2013). However, it was shown that the mutation of the *rsmN* gene did not abolish swarming motility, but rather decreased it. Investigating the effect of a double *rsmArsmN* mutant on swarming motility remains to be further studied. However, since the only known major target of both *rsmY* and *rsmZ* is the RsmA post-transcriptional repressor (Brencic and Lory, 2009), our data strongly suggest that these sRNA have alternative targets, yet to be identified.

As there is increasing evidence that sRNA control by HptB can be due to the alternative PA3346/PA3347 regulation pathway and to understand how that phosphotransfer protein can have an effect of sRNA regulation, we created a double Δ*hptBgacA* mutant. Knowing that GacA is the main positive regulator of *rsmY* and *rsmZ* expression (Brencic et al., 2009), we expected to see a loss of sRNA upregulation in that double mutant. As anticipated, we observed a downregulation of both *rsmY* and *rsmZ* in a planktonic culture of the Δ*gacA* mutant as it has already been reported by other groups (Kay et al., 2006; Brencic and Lory, 2009) as well as in swarming cells of that same genotype (**Figure 4**). Furthermore, the introduction of an *hptB* deletion in the Δ*gacA* mutant strain resulted in the abolishment of both *rsmY* and *rsmZ* upregulation that was observed in the simple Δ*hptB* mutant in liquid cultures. Interestingly, Bordi et al. (2010) observed that the hyperbiofilm phenotype of a PAKΔ*hptB* strain was indeed abolished in a double Δ*hptBgacA* mutant suggesting that both sRNAs are implicated in the control of such a phenotype in strain PAK. Surprisingly in swarming cells, we observed that *rsmZ* was still upregulated when compared to a simple Δ*gacA* mutant whereas *rsmY* overexpression was lost in a double Δ*hptBgacA* mutant. This result reveals a differential regulation of both sRNAs in swarming cells, where *rsmZ* transcription does not require the presence of GacA as it has been seen for planktonic cultures, but rather necessitates the contribution of unknown regulator(s) that are specifically active in surface-grown cells.

In addition of GacA being the main activator of *rsmY* and *rsmZ* transcription (Brencic et al., 2009), there are other global regulators that can influence the expression levels of these sRNAs in planktonic cultures. Accordingly, the DNA-binding global negative regulators MvaT and MvaU, can specifically repress *rsmZ* expression without affecting *rsmY* (Castang et al., 2008) thus possibly being the reason why the upregulation was observed in surface-grown cells. We assessed the swarming motility of both Δ*mvaT* and Δ*mvaU* mutant strains and did not observe any difference in surface movement when compared to the wild-type (Supplementary Figure S6), indicating that any effect on *rsmZ* regulation does not strictly exert an output on swarming motility.

To rule out the possibility of sRNA regulation by the PA3346/PA3347 pathway, we monitored the expression of *rsmY* and *rsmZ* in both swarming cells and their planktonic counterpart of mutants in these genes. Opposite to Bordi et al. (2010), we observed a slight increase in *rsmY* expression in the *PA3346^-^* mutant background but no effect for both sRNAs in *PA3347^-^* in planktonic cultures (Supplementary Figure S7). Also, the inactivation of either *PA3346* or *PA3347* did not affect *rsmY* and *rsmZ* expression in surface-grown cells (Supplementary Figure S8). These results indicated us that *rsmZ* overexpression in a Δ*hptB* genetic mutant background is somehow independent of both GacS/GacA and PA3346/47 regulation pathways and that an unknown surface-activated regulator is responsible for the observed effect. Recently, a study by Wang et al. (2014), characterized a novel swarming regulator, BswR, capable of controlling the expression of *rsmZ* directly in PAO1. We verified whether BswR could be responsible for *rsmZ* regulation in swarming cells, but, surprisingly, we did not observe any effect on *rsmZ* expression in a Δ*bswR* mutant in both planktonic and swarming cells (Supplementary Figure S9), indicating that BswR does not play a role in the regulation of this sRNA under our conditions. Earlier this year, a study published by Xu et al. (2016) characterized the HapZ adaptor protein in a PAO1 strain and observed that it could act as an intermediate between the membrane sensor SagS and the HptB protein (Xu et al., 2016). However, it was reported that a mutation in the *sagS* gene does not affect swarming motility in PA14 (Petrova and Sauer, 2011), making HapZ unlikely to participate in swarming motility regulation, at least, in a PA14 genetic background.

**FIGURE 5:**
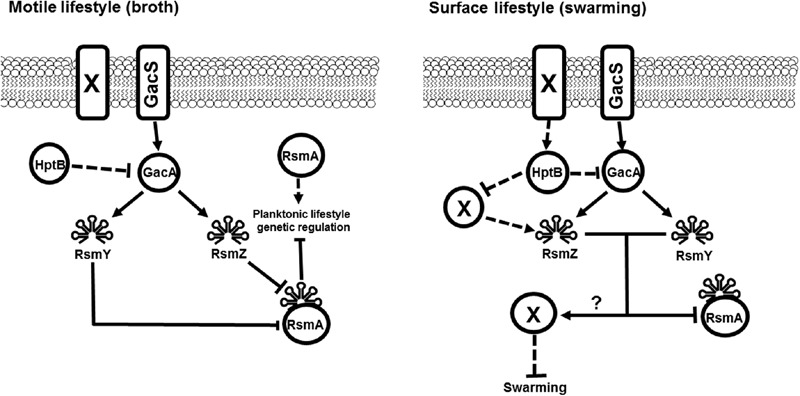
**Model for broth-surface differential sRNA regulation.** We propose a regulation model where the control of the expression of *rsmY* and *rsmZ* by HptB is under the exclusive control of the GacS/GacA system and converges to the post-transcriptional regulator RsmA for both planktonic and swarming cells. However, in cells grown on a surface such as in swarming motility, the regulation of both *rsmY* and *rsmZ* is differential and does not strictly mediate their output *via* RsmA. The obtained data in our study indicates that other key-players allows for regulation of *rsmZ* by HptB and does not implicate the Gac system. This regulation most likely involves other membrane sensors that can modulate the activity of the HptB protein. Presented model integrates previously published data (Hsu et al., 2008; Brencic et al., 2009; Bordi et al., 2010). Full arrows represent direct positive regulation. Dashed arrows represent indirect positive regulation. Dashed bars represent indirect negative control. Full bars represent direct negative regulation. ? = unknown contribution. X = Unknown regulating factor.

The discrepancies observed between our study and other ones (Bordi et al., 2010; Bhuwan et al., 2012; Wang et al., 2014) stress out the growing evidence that there are regulatory differences between the various *P. aeruginosa* strains (Tremblay and Déziel, 2008; Mikkelsen et al., 2011). Furthermore, we certainly cannot rule out the implication of the numerous previously characterized elements found to be implicated in the regulation of swarming motility. However, here we present a model where small RNA regulation is dependent on the conditions in which we study *P. aeruginosa* (i.e., broth *versus* surface) which favor two different bacterial lifestyles. Furthermore, RsmW a novel RNA under the control of the Gac system has been identified to be strongly upregulated in biofilm conditions compared to planktonic cultures which further strengthens the existence of differential genetic regulation depending on the selected growth conditions (Miller et al., 2016). Our data confirm that the GacS/GacA system is not the only one responsible for the control of *rsmZ* expression and implicates the presence of unidentified key factors that are important when cells are grown on a surface rather than in planktonic cultures, as exemplified by the use of swarming motility as a model for surface behavior (**Figure 5**). Further downstream in the swarming regulatory cascade, we also show evidence that RsmY and RsmZ can modulate swarming motility not only via RsmA inhibition. Further experiments focusing on identifying these regulatory players specific to surface-grown bacteria will unveil a whole misunderstood genetic regulation portrait of *P. aeruginosa*.

## Author Contributions

FJ-P, JT, and ED conceived and designed the experiments. FJ-P and JT performed the experiments. FJ-P, JT, and ED analyzed the data. FJ-P, JT, and ED contributed to manuscript preparation and editing.

## Conflict of Interest Statement

The authors declare that the research was conducted in the absence of any commercial or financial relationships that could be construed as a potential conflict of interest.

## Footnotes

1 http://frodo.wi.mit.edu/primer3/
