# Supplementary material for: Broth versus Surface-Grown Cells: Differential Regulation of RsmY/Z Small RNAs in Pseudomonas aeruginosa by the Gac/HptB System
Source: Front Microbiol. 2017 Jan 10;7:2168. doi: 10.3389/fmicb.2016.02168 (PMC5222819; doi:10.3389/fmicb.2016.02168)
Supplement: Supplementary file 1 [file Data_Sheet_1.PDF]

Supplementary material

Broth versus surface-grown cells: Differential regulation of RsmY/Z small RNAs by HptB in *Pseudomonas aeruginosa* by the Gac/HptB system

Fabrice Jean-Pierre[1], Julien Tremblay[1,2] & Eric Déziel[1*]

[1]INRS-Institut Armand-Frappier, 531 Boul. Des Prairies, Laval, Québec, Canada

[2]National Research Council Canada, Montréal, Canada

*For correspondence. E-mail: eric.deziel@iaf.inrs.ca, Tel. 450-687-5010

Supporting experimental procedures

Congo Red assay

Tryptone (1%) agar plates were supplemented with 80 µg ml^{-1} Congo Red and 20 µg ml^{-1} Coomassie brilliant blue solidified with 0.5% agar (Bacto) were inoculated with 5 µl of bacterial suspension diluted to OD_{600} = 0.05 and incubated at room temperature for 7 days. Experiment was done using three replicates on two different days. Shown is typical EPS production phenotype.

β-galactosidase assays

Activity of *lacZ* fusion reporters was tested for β-galactosidase activity with *o*-nitrophenyl-β-D-galactopyranoside (ONPG, Thermo Fisher Scientific, Nepean, ON, Canada) as substrate (Miller, 1972). Each experiment was performed using three biological replicates. Overnight TSB cultures were diluted at a starting OD_{600} of 0.05 in M9DCAA and incubated at 34°C. Results were obtained for five sampling points during bacterial growth over 8 hours.

After 12 hours incubation at 34°C the cells located at the extremity of three separate tendrils of a swarming strain were collected. The tendrils were resuspended in 100 µl of 1X PBS and vortexed thoroughly. The OD_{600} was measured using a Nanodrop ND-1000 and adjusted to an OD_{600} = 0.1. β-galactosidase activity was determined as described above.

Static biofilm formation

A 500 µl bacterial suspension volume at OD_{600} = 0.05 was cultivated statically in a 5 mL polystyrene tube at 34°C during various sampling times in M9DCAA medium. For analysis, the tube was vigorously rinsed by milli-Q water then incubated 10 minutes with 1 mL of 1% crystal violet at room temperature then rinsed again with milli-Q water. The colorized biofilm ring was then solubilized with 4 mL of 95% ethanol then vortexed with glass beads. OD_{595} was measured using a spectrophotometer. The experiment were performed using three technical replicates at least three times.

Creation of a Δ*hptBgacA* mutant by lambda-red recombinase

The mutated *gacA* gene was amplified from the *gacA*::MaR2xT7 ID34781 PA14 non-redundant set with the primers listed in Table S1. The Δ*hptB* mutant containing the pUCP18-RedS was grown in TSB supplemented with 300 µg ml^{-1} carbenicillin to an OD_{600} = 0.5 at 37°C. Then, the plasmid was induced for 3 hrs with 0.2% L-arabinose. After induction, the cells were washed four times with 1 ml of 10% sucrose and concentrated to a final volume of 100 µl. A concentration of 5 µg the gel-purified *gacA*::Mar2xT7 gene amplification was electroporated and incubated in 1 ml TSB for 2 hrs at 37°C. Transformants were selected by plating electroporated cells on TSB agar plates supplemented with 300 µg ml^{-1} and incubated at 37°C until colonies were visible. The selected colonies were further verified by PCR for correct insertion using the PA14_gacA_For_FJP + FJP_PA14_gacA_Rev and PA3345_Left_FWD_HindIII + PA3345_Right_REV_SmaI primer sets.

Supporting figures

Table S1 Primers used in this study

Mutagenesis primers (5' → 3')	
**Name**	**Primer sequence**
PA3345_Left_FWD_HindIII	ccc**aagctt**gggTGCGGGTCGAGGACAGCGG
PA3345_Left_REV	*ttctatcgttcgcta*GAGATGCGGCGCGGACATTC
PA3345_Right_FWD	*tagcgaacgatagaa*CGCCTGCGCAGCCTGCAT
PA3345_Right_REV_SmaI	tcc**cccggg**ggaTACGCCAGGGAGGCTCGA
PA14_gacA_For_FJP	TCGGCGATGGTCGCTATG
FJP_PA14_gacA_Rev	TAGCGAGGAAGGCGCTCGC
RT-PCR primers	
rsmZq_fwd	GAACACGCAACCCCGAAG
rsmZq_rev	CCACTCTTCAGTCCCTCGTC
rsmYq_fwd	AGGAAGCGCCAAAGACAATA
rsmYq_few	GGGTTTTGCAGACCTCTATCC
nadBq_fwd[1]	CTACCTGGACATCAGCCACA
nadBq_rev[1]	GGTAATGTCGATGCCGAAGT

*In bold = restriction sites. In italics = overlapping sequences to *hptB* gene

[1]*nadB* housekeeping gene qRT-PCR primers from (Tremblay and Deziel, 2010)

Table S2 Strains used in this study

Strains/Plasmids	ED #	Phenotype/Genotype	Reference
**Strains**			
PA14	14	UCBPP-PA14 wild-type strain	(Rahme et al., 1995)
Δ*hptB*	1214	Markerless *hptB* deletion	This study
mvaT$^-$	289	*MrT7* transposition insertion mutant, Gm^R, ID34492	(Liberati et al., 2006)
mvaU$^-$	806	*MrT7* transposition insertion mutant, Gm^R, ID42058	(Liberati et al., 2006)
rsmA$^-$	282	*MrT7* transposition insertion mutant, Gm^R, ID44507	(Liberati et al., 2006)
PA3346$^-$	1260	*MrT7* transposition insertion mutant, Gm^R, ID31270	(Liberati et al., 2006)
PA3347$^-$	1261	*MrT7* transposition insertion mutant, Gm^R, ID39911	(Liberati et al., 2006)
bswR$^-$	2681	*MrT7* transposition insertion mutant, Gm^R, ID24728	(Liberati et al., 2006)
**Plasmids**			
pUCP18-RedS		Arabinose-inducible recombination plasmid, Cb^R	(Lesic and Rahme, 2008)
pCTX-*rsmY*-*lacZ*		Self-proficient integration vector with *lacZ* reporter for *rsmY* transcriptional expression	(Brencic and Lory, 2009)
pCTX-*rsmZ*-*lacZ*		Self-proficient integration vector with *lacZ* reporter for *rsmZ* transcriptional expression	(Brencic et al., 2009)

Supporting figures

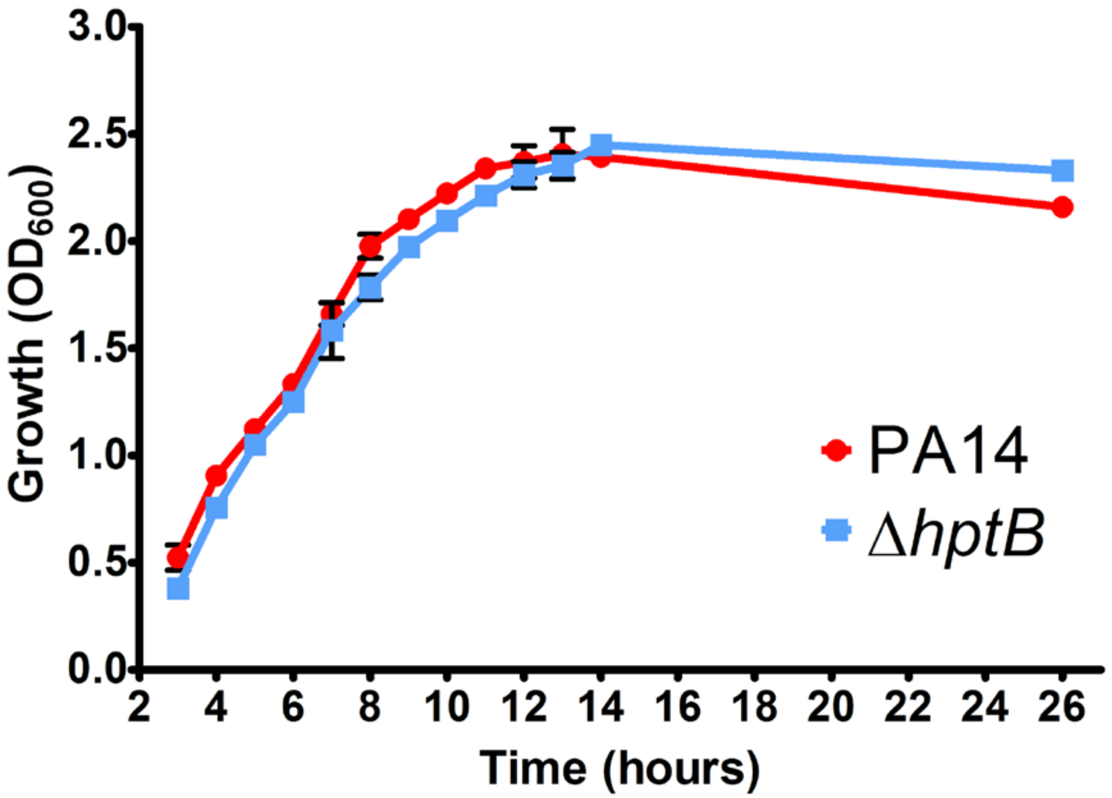

Figure S1: Growth curve of the PA14 and Δ*hptB* strain in M9DCAA at 34°C. Data represents the average of three technical replicates. Error bars represent the standard deviation. Experiment was repeated at least twice.

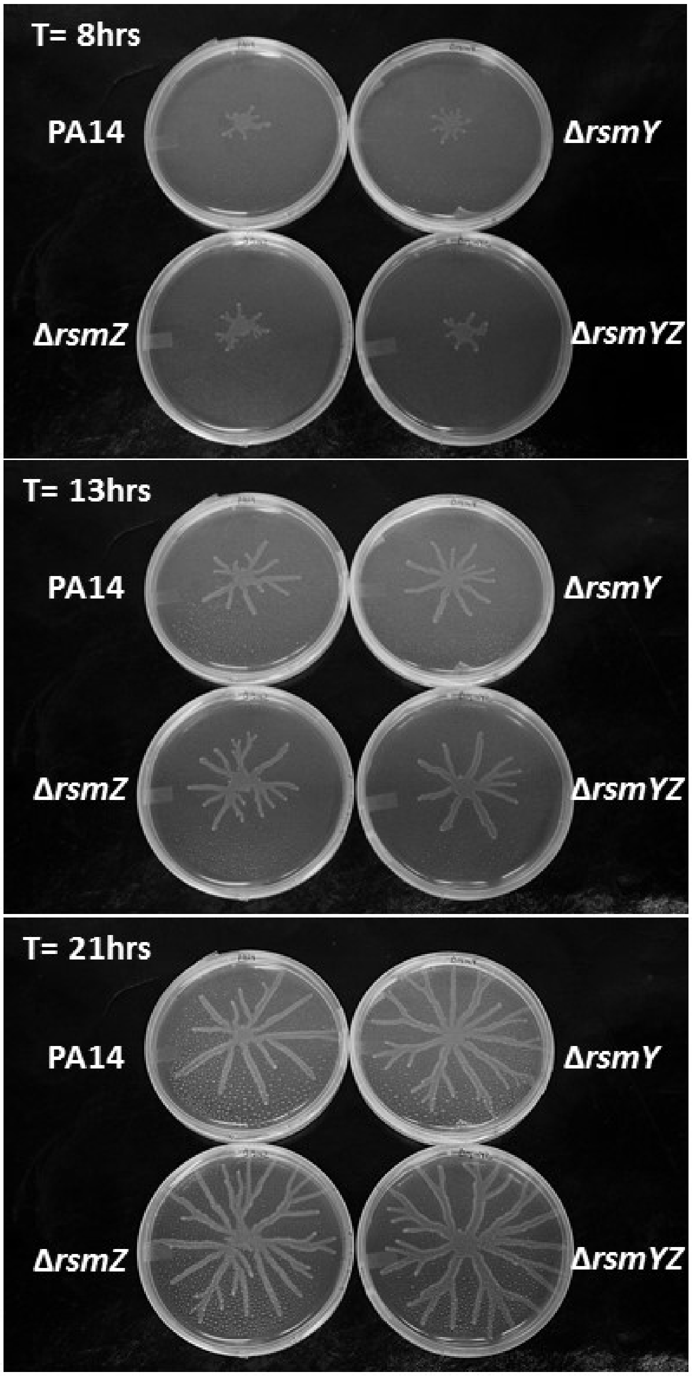

Figure S2: Endpoint pictures of the Δ*rsmY/Z* mutants time-lapse analysis at different time points.

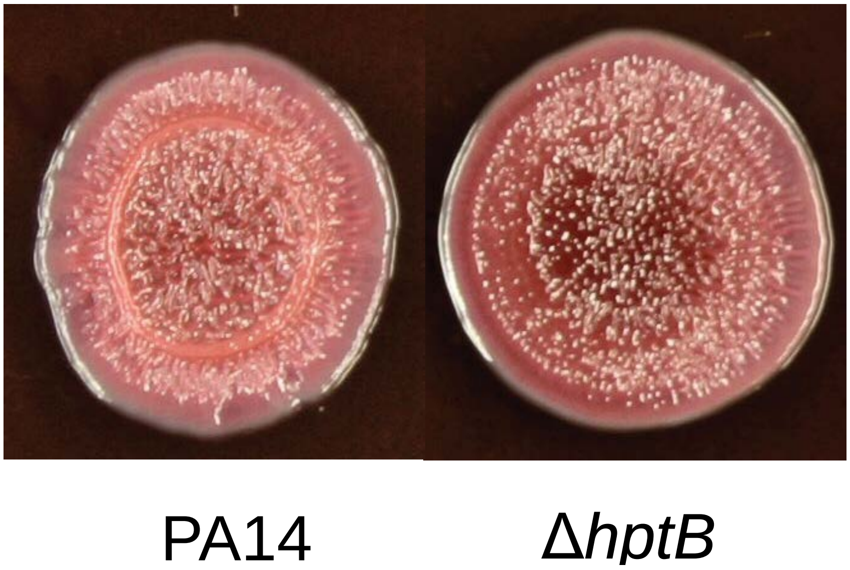

Figure S3: Exopolysaccharide fixation determined by Congo Red assay.

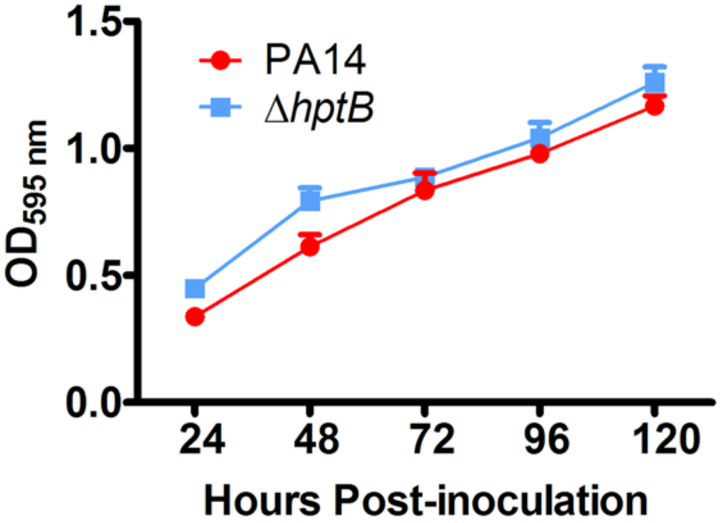

Figure S4: Biofilm formation assay of the PA14 and Δ*hptB* strains. Data represents the average of three replicates. Error bars represent the standard deviation. Experiment was repeated at least twice.

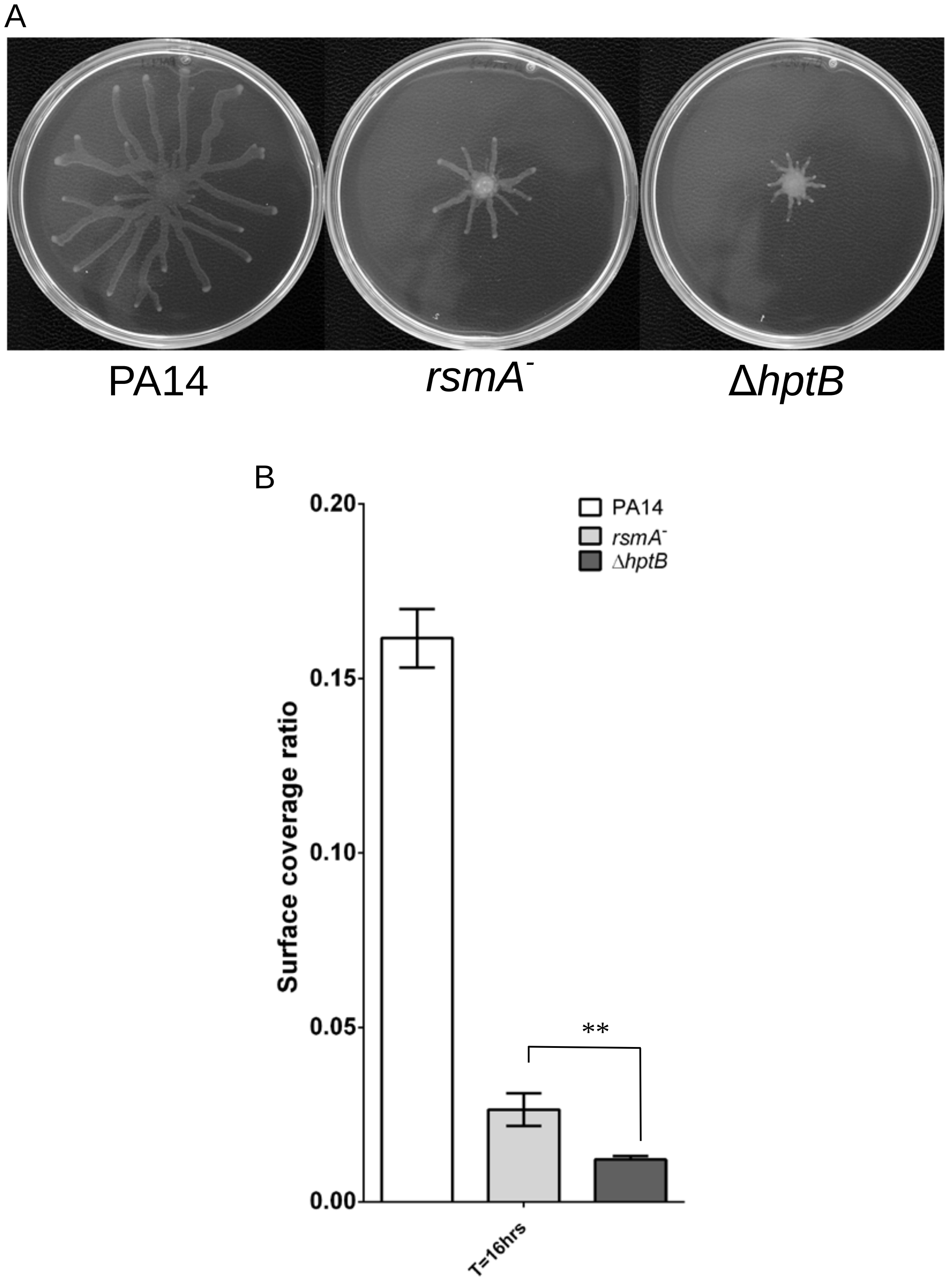

Figure S5: Various swarming phenotype. (A) Swarming motility of the PA14, *rsmA*$^-$ and Δ*hptB* strains. (B) Surface coverage of the PA14, *rsmA*$^-$ and Δ*hptB* strains. Data represents the average of three technical replicates. Error bars represent the standard deviation of the three technical replicates. Experiment was repeated at least twice. Statistical Student's *t*-test analysis was based on two independent experiments (**, $p < 0.01$).

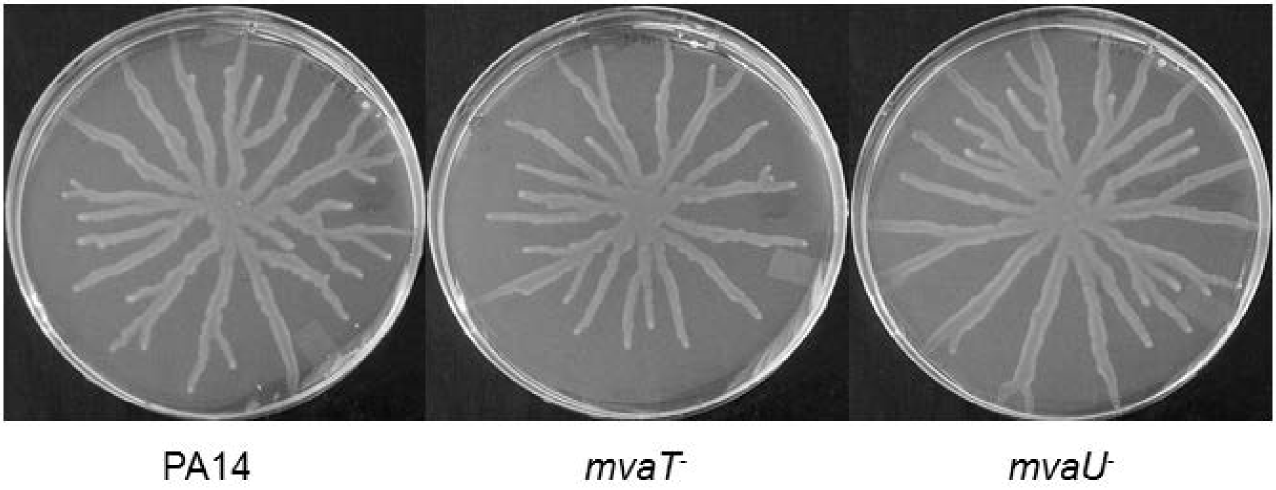

Figure S6: Swarming phenotypes of the PA14, *mvaT*⁻ and *mvaU*⁻ strains.

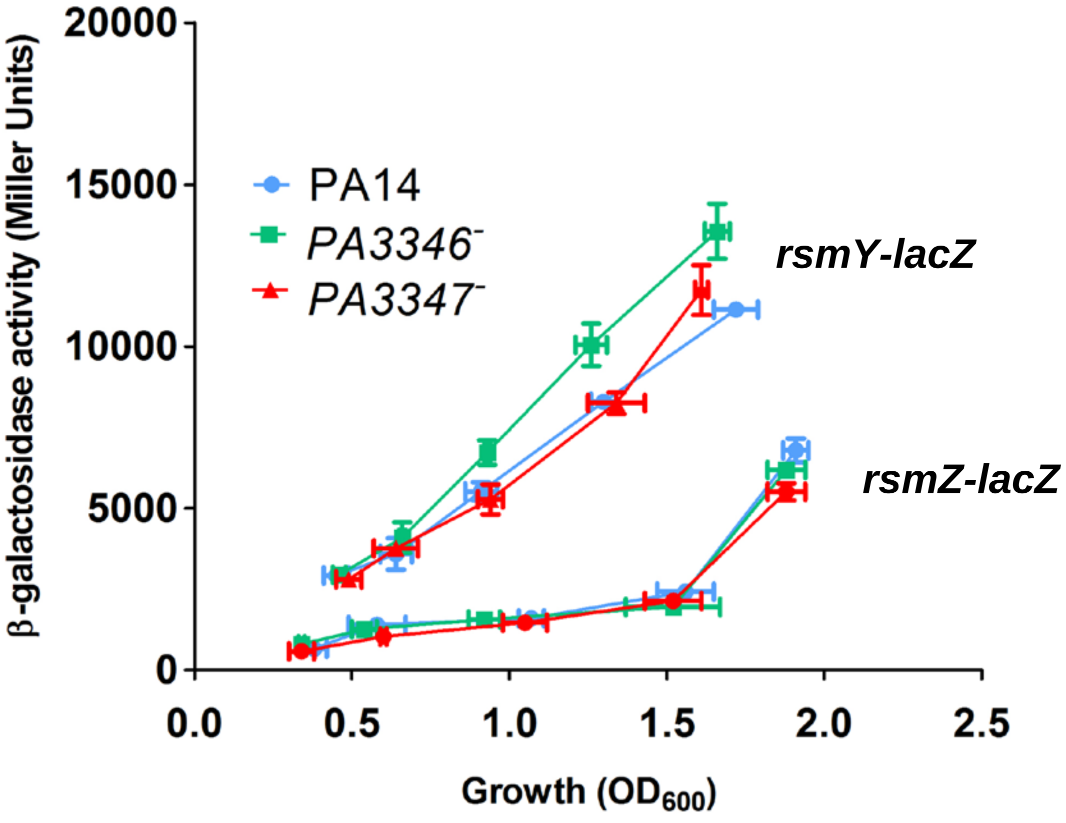

Fig S7: Time-course of *rsmY-lacZ* and *rsmZ-lacZ* in various genetic backgrounds grown in M9DCAA broth over 8 hours. Data represents the average of three biological replicates. Error bars represent the standard deviation.

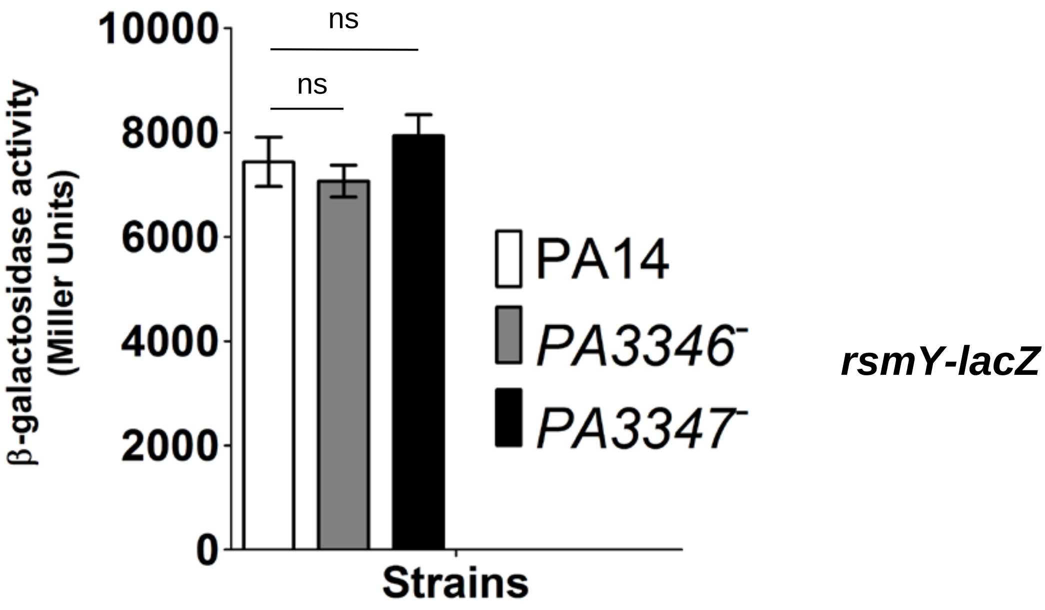

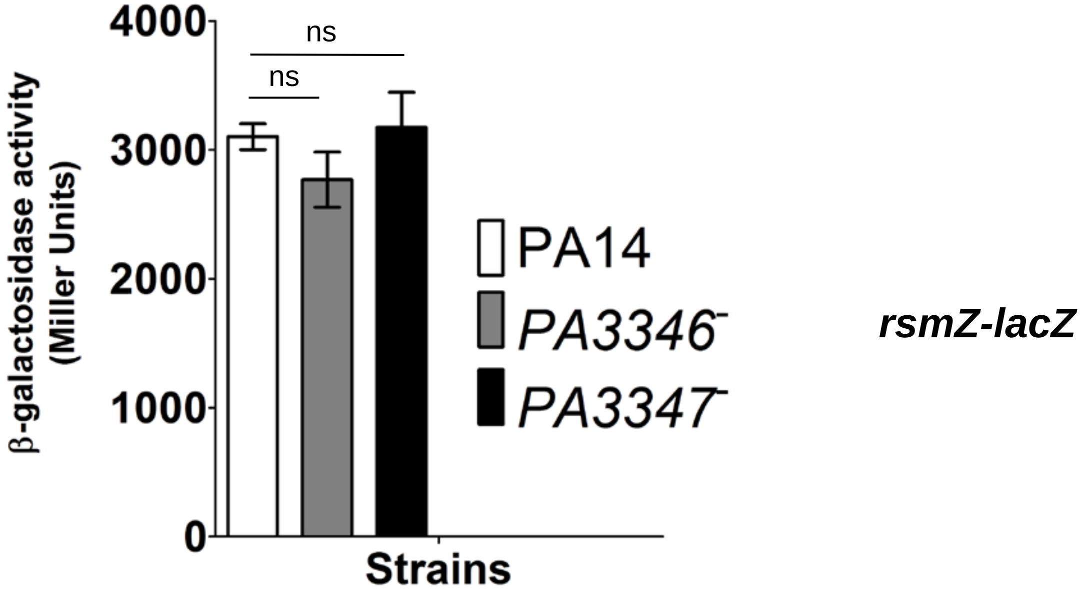

Fig S8: Time-course of *rsmY-lacZ* and *rsmZ-lacZ* in various genetic backgrounds grown as swarming colonies on M9DCAA. Data represents the average of three biological replicates. Error bars represent the standard deviation. Statistical Student's *t*-test analysis was performed with ns = not significant.

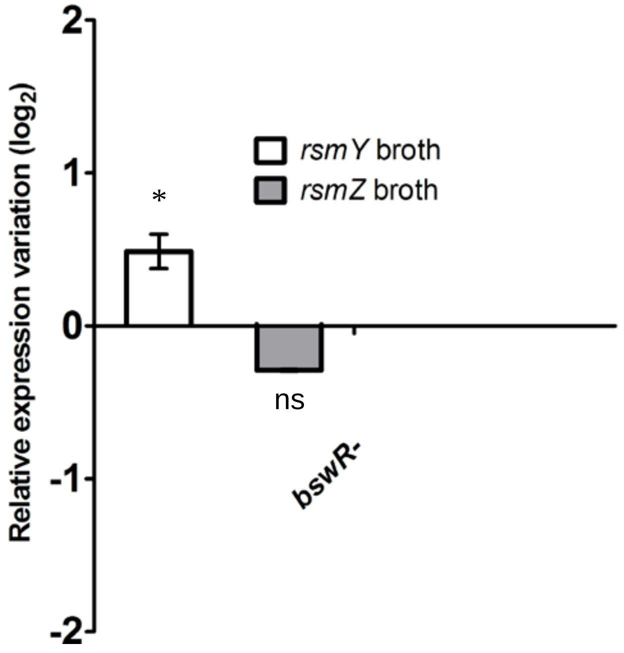

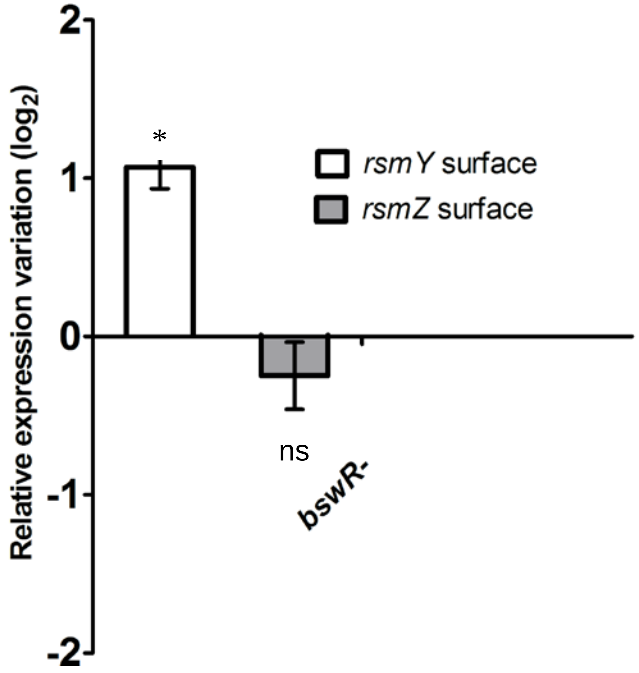

Figure S9: qRT-PCR on the Δ*bswR* mutant grown in M9DCAA broth and swarming conditions. Data represents the average of three biological replicates. Error bars represent the standard deviation of three biological replicates. Statistical Student's *t*-test analysis was performed on two independent experiments with *, $p < 0.05$, ns = not significant.

<u>**References**</u>

Brencic, A., and Lory, S. (2009). Determination of the regulon and identification of novel mRNA targets of *Pseudomonas aeruginosa* RsmA. *Mol Microbiol* 72**,** 612-632. doi: 10.1111/j.1365-2958.2009.06670.x.

Brencic, A., Mcfarland, K.A., Mcmanus, H.R., Castang, S., Mogno, I., Dove, S.L., and Lory, S. (2009). The GacS/GacA signal transduction system of *Pseudomonas aeruginosa* acts exclusively through its control over the transcription of the RsmY and RsmZ regulatory small RNAs. *Mol Microbiol* 73**,** 434-445. doi: 10.1111/j.1365-2958.2009.06782.x.

Lesic, B., and Rahme, L.G. (2008). Use of the lambda Red recombinase system to rapidly generate mutants in *Pseudomonas aeruginosa*. *BMC Mol Biol* 9**,** 20. doi: 10.1186/1471-2199-9-20.

Liberati, N.T., Urbach, J.M., Miyata, S., Lee, D.G., Drenkard, E., Wu, G., Villanueva, J., Wei, T., and Ausubel, F.M. (2006). An ordered, nonredundant library of *Pseudomonas aeruginosa* strain PA14 transposon insertion mutants. *Proc Natl Acad Sci U S A* 103**,** 2833-2838. doi: 10.1073/pnas.0511100103.

Miller, J.H. (1972). *Experiments in molecular genetics.* Cold Spring Harbor, N.Y.: Cold Spring Harbor Laboratory.

Rahme, L.G., Stevens, E.J., Wolfort, S.F., Shao, J., Tompkins, R.G., and Ausubel, F.M. (1995). Common virulence factors for bacterial pathogenicity in plants and animals. *Science* 268**,** 1899-1902.

Tremblay, J., and Deziel, E. (2010). Gene expression in *Pseudomonas aeruginosa* swarming motility. *BMC Genomics* 11**,** 587. doi: 10.1186/1471-2164-11-587.
